# Evaluation of Salmon, Tuna, and Beef Freshness Using a Portable Spectrometer

**DOI:** 10.3390/s20154299

**Published:** 2020-08-01

**Authors:** Eui Jung Moon, Youngsik Kim, Yu Xu, Yeul Na, Amato J. Giaccia, Jae Hyung Lee

**Affiliations:** 1 Department of Radiation Oncology, Stanford University, Stanford, CA 94305, USA; ejung12@stanford.edu (E.J.M.); yxu33@stanford.edu (Y.X.); giaccia@stanford.edu (A.J.G.); 2 Stratio Inc., Palo Alto, CA 94303, USA; youngsik.kim@stratiotechnology.com (Y.K.); yeul.na@stratiotechnology.com (Y.N.)

**Keywords:** food freshness, portable spectrometer, near-infrared, machine learning

## Abstract

There has been strong demand for the development of an accurate but simple method to assess the freshness of food. In this study, we demonstrated a system to determine food freshness by analyzing the spectral response from a portable visible/near-infrared (VIS/NIR) spectrometer using the Convolutional Neural Network (CNN)-based machine learning algorithm. Spectral response data from salmon, tuna, and beef incubated at 25 °C were obtained every minute for 30 h and then categorized into three states of “fresh”, “likely spoiled”, and “spoiled” based on time and pH. Using the obtained spectral data, a CNN-based machine learning algorithm was built to evaluate the freshness of experimental objects. In addition, a CNN-based machine learning algorithm with a shift-invariant feature can minimize the effect of the variation caused using multiple devices in a real environment. The accuracy of the obtained machine learning model based on the spectral data in predicting the freshness was approximately 85% for salmon, 88% for tuna, and 92% for beef. Therefore, our study demonstrates the practicality of a portable spectrometer in food freshness assessment.

## 1. Introduction

Freshness is one of the most important parameters to evaluate food quality, especially for raw food such as fish and meat. A majority of grocery stores and/or consumers rely on sensory methods such as appearance, odor, taste, or texture to assess food freshness. However, these methods are often both subjective and inaccurate, as they are heavily dependent on the skill level of the inspector.

Although qualitative measurement for freshness is more reliable and precise, it requires expensive, slow, and/or destructive methods [1,2]. Physicochemical methods measure water content, pH value, thiobarbituric acid reacted substance (TBARS), total volatile basic nitrogen (TVB-N), sugar, and nutrition, including vitamins. Although these methods are widely used in controlled environments such as large-scale food processing plants, they are either destructive or prone to cross-contamination. Microbiological methods provide useful information regarding bacterial spoilage, but accurate measurement takes time. Therefore, alternative methods are required for grocery stores and/or consumers, who are in need of prompt results for quality assessment. Most recently, a non-destructive and in situ analysis for a fish freshness monitoring system was proposed by a group of researchers using the optical pH sensor; however, the absolute pH values do not necessarily increase or decrease monotonically during the testing period, which make it challenging to assess the freshness without monitoring the whole period [3].

Freshness assessment using a visible/near-infrared (VIS/NIR) spectrometer has been proposed by previous studies [4,5]. However, this approach has several drawbacks, as most of the well-known spectrometers are both bulky and expensive. Moreover, due to unique spectral response features of each food, the data need to be analyzed in different ways depending on food types. More recently, a breakthrough in technology enabled the production of low-cost spectrometer on a “chip”, leading to the development of consumer-scale NIR devices including LinkSquare [6,7,8].

**Figure 1:**
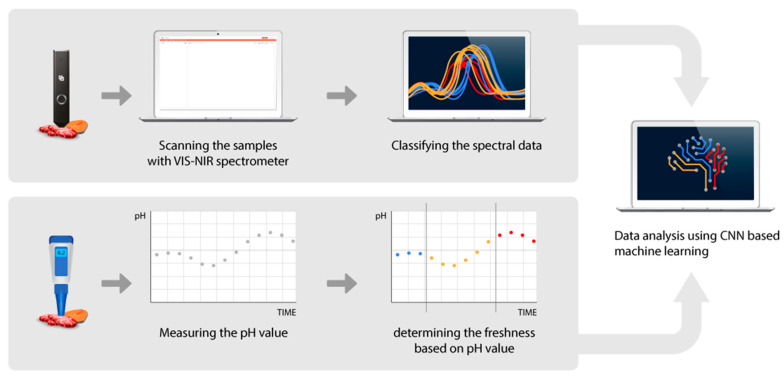
Overview of the food freshness estimation process using a portable visible/near-infrared (VIS-NIR) spectrometer, LinkSquare. As the rotting process of the sample continues, the spectral responses are collected over time with a LinkSquare device (above), and the pH values are measured at the same time (below) to determine the freshness of the samples. The spectral response data is used to train a machine learning model for the food freshness estimation (right).

In this study, we introduce a smart method using LinkSquare, which is a handheld spectrometer that was designed and built by ourselves and commercially available for sale to the public [7,8,9,10,11,12], to assess food freshness (Figure 1). Samples were scanned with a VIS/NIR portable spectrometer and spectral data was analyzed using the Convolutional Neural Network (CNN)-based machine learning algorithm to evaluate the freshness of the objects [13,14]. The conventional pH meter was used to categorize the freshness and to verify our results.

## 2. Materials and Methods

### 2.1. Instruments

A PH60F pH meter (Apera Instruments, Columbus, OH, USA), which ranges from 2.00 to 16.00 pH with an accuracy of ±0.01 pH, was used for the pH measurement. 

LinkSquare^®^ (www.linksquare.io), a portable VIS/NIR spectrometer, was used for the spectral response measurement. It was equipped with optical parts, an image sensor, a microcontroller unit (MCU), a white light-emitting diode (LED), a bulb, and a rechargeable Li-ion battery. The dimensions of the device were as small as 114.0 × 23.9 × 23.9 mm, and its weight was 57 g. The spectral range of the spectrometer was 400 to 1000 nm with a resolution of 5 to 30 nm (approximately 5 nm @ 500 nm, 10 nm @ 700 nm, and 20 nm @ 900 nm). Since each spectrometer unit was factory calibrated, data obtained from one unit can be applied to others with minimum error. These data will be called ‘spectral response data’ henceforth.

An AI Platform (ai.linksquare.io) to analyze the spectral response data using a CNN-based machine learning algorithm was developed by Stratio, Inc. (www.stratiotechnology.com). 

### 2.2. Sample Preparation and Experiment Setup

The Atlantic salmon (Salmon salar) and Pacific salmon (Oncorhynchus nerka) were fresh and unfrozen, while tuna was frozen and imported from Indonesia. For beef, choice-grade USDA sirloin beef was used.

Each sample was sliced into pieces with the size of 30 × 30 × 30 mm to be clamped together with a portable spectrometer by using a custom-built holder inside an incubator that maintains the temperature at 25 °C to make a portable spectrometer direct contact to each food sample. Samples were placed on a glass container, and a spectrometer was installed on top of each sample, directly contacting the surface. A spectrometer emitted light toward the surface of a sample and measured the reflected light spectrum. Spectral response data were automatically recorded every minute and stored with its timestamp. More than 15 samples for each food and 9 devices were used in this study (Table 1). The spectra of 3 Atlantic salmon, 3 Pacific salmon, 8 tuna, and 8 beef samples were scanned for creating a training dataset, while the spectra of 12 Atlantic salmon, 12 Pacific salmon, 9 tuna, and 8 beef samples were scanned for a verification dataset.

To track the freshness, an additional 3 samples per sample for the spectrum measurement were prepared for the pH measurement. These extra samples underwent the same environment setup in the incubator. At the end of each period, which varied from one hour to five hours, a small (approximately 2 g) piece was sliced out of each sample, and pH values were measured 20 times to minimize the measurement error. From these 20 measurements, the mean and the standard deviation were calculated.

### 2.3. Staphylococcus Aureus Culture

Staphylococcus aureus was isolated from food samples following protocols from the previous publication [15,16]. Meat and fish samples were cut into 5 cm × 5 cm patches, which weigh approximately 50 g, and incubated for 0, 6–8, and 24 h. Samples were hand massaged with 10 mL of buffered peptone water (BPW, Simga-Aldrich) for 5 min and only fluid was collected using cheesecloth wipes (VWR). Then, 100 μL of extracts were plated on Baird Parker (BP) selective media plates (Millipore Sigma) and incubated for 24 h at 37 °C. To count colonies, plate images were taken and analyzed using a customized python script (python 3.7, Wilimington, DE, USA) based on color thresholding and object size. The number of colonies was further validated by visual examination.

### 2.4. TBARS Assay

First, 2 g of food samples were submerged in 5 mL of ice cold 10% trichloracetic acid (TCA) and incubated for 5 min on ice. Samples were homogenized in ice and spun down for 5 min at 4000 rpm. Supernatants were transferred and then spun down again for 5 min at 14,000 rpm. Supernatants were mixed with TBA reagents from a TBARS assay kit (BioAssay System) and incubated for 60 min at 100 °C. Optical density (OD) values were measured at 535 nm using a plate reader.

### 2.5. CNN-Based Machine Learning Algorithm 

A CNN structure with 6 sets of convolution layers, rectified linear unit (ReLU) layers, and pooling layers followed by three fully connected layers was used in this study. Since the input was 1-Dimensional (D) instead of 2-D in the typical computer vision problem, a small modification was made on the convolution layers so that they perform only 1-D convolution. Except for the small modification, the neural network architecture used in this study is similar with that of AlexNet^16^. AlexNet with a shift-invariant feature can minimize the effect of the variation caused using multiple devices in a real environment. A machine learning model for each tested food category was made, which gives four models in total for this study.

The spectral response data that cover the full spectral range (400–1000 nm) were provided as input vectors of the CNN structure. Two light sources, an LED and a bulb, were used in LinkSquare^®^, which is why one set of inputs consisted of two 1-D spectral response vectors (600 × 1). They were treated separately through the modified CNN structure, which consists of 1-D convolution layers and 1-D max pooling layers until the last pooling layer, and these were combined together to form a 1-D input vector of the first fully connected layer. Three categories (“fresh”, “likely spoiled”, and “spoiled”) for each food type were properly encoded and were provided as output vectors of the last fully connected layer.

The training of the CNN-based machine learning algorithm was implemented using the Torch framework on a NVidia GeForce GTX Titan X. For each experimental setup, several runs of experiments were performed independently. Some of the runs became the training dataset, and the rest of them became a completely separated dataset for testing, which was also known as the verification dataset. The training dataset was divided into two smaller sets: 90% of them were used for actual training, while 10% of them were only for checking the current performance of the model by evaluating the accuracy. The division was done randomly among the scans from various samples and devices. The accuracy was calculated only using the aforementioned verification dataset.

### 2.6. Sensitivity and Specificity

The values of sensitivity and specificity are determined using the following equations:

(1) $$\mathrm{Sensitivity}=\frac{\mathrm{TP}}{\mathrm{TP}+\mathrm{FN}}$$

(2) $$\mathrm{Specificity}=\frac{\mathrm{TN}}{\mathrm{FP}+\mathrm{TN}}$$

The concepts of True Positive (TP), True Negative (TN), False Positive (FP), and False Negative (FN) in the above equations can be easily defined with a binary classification test.

## 3. Results and Discussion

### 3.1. ph Values to Determine Food Freshness

Changes in pH values on the surface of the beef and fish samples are useful in tracking the freshness of food samples [17,18,19]. The accumulation of lactic acid after post mortem glycolysis causes a decrease in pH. After this initial phase, pH increases as volatile basic amines are produced from the spoilage of food [20,21]. Thus, to determine time points of food spoilage, we measured pH on food samples as an indicator of freshness.

**Figure 2:**
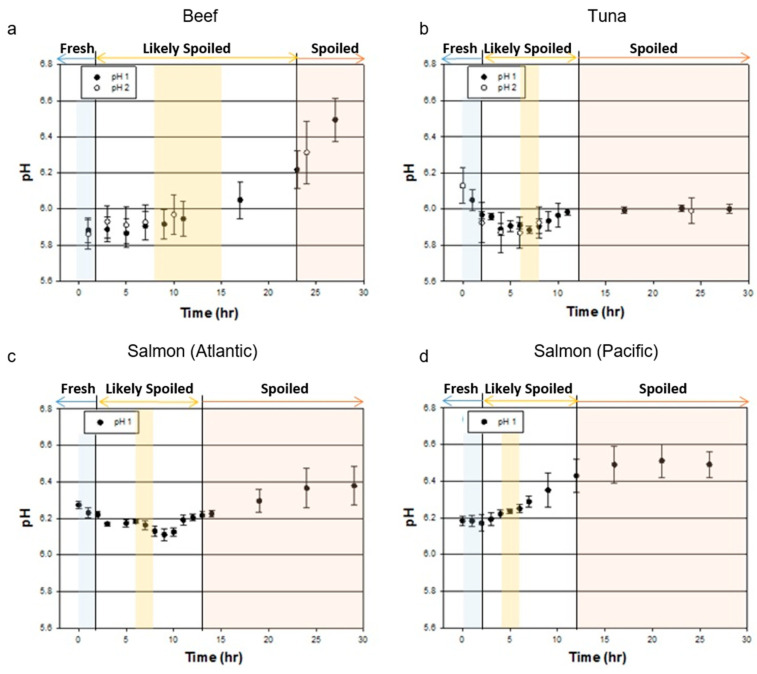
Time series of pH values for food samples to determine freshness: (**a**) beef, (**b**) tuna, (**c**) Atlantic salmon, and (**d**) Pacific salmon. At each time slot, the data point is for the mean of 60 measurements (3 sample × 20 measurements), and the bar represents the standard deviation of them. There were two separate rotting processes in the beef and tuna experiments, which are presented as pH1 and pH2. Spectral response data collected during the shaded time slots (blue, yellow, and red) were used for the machine learning model.

In beef, pH ranging from 5.7 to 6.0 is considered to be fresh, while meat with a pH higher than 6.2 refers to dark, firm, and dry (DFD) meat, which is susceptible to the growth of microorganisms [22,23]. As shown in Figure 2a, the pH of our beef sample increased from 5.9 to 6.5 during the 30-h period of measurement. We identified beef in the “fresh” state during the initial 2-h period when its pH was lower than 6.0. After a 23-h incubation, the pH became higher than 6.2, and we categorized the beef’s state as “spoiled”. We termed beef as “likely spoiled” between 2 and 23 h when pH values were 6.0–6.2. 

In fresh tuna, pH is between 5.2 and 6.1, while pH is between 6.1 and 6.3 in fresh salmon [24,25,26]. Unlike beef, fish is frozen immediately after catching, which is why we observed decreases in pH in most of our fish samples during initial measurements. In tuna, pH decreased from 6.1 to 5.9 during the first 7-h measurement. Then, the pH value increased to 6.0 for 4 h and remained at pH 6.0 until the end of the measurement (Figure 2b). 

In Atlantic salmon, the pH decreased from 6.3 to 6.1 during the first 9 h of the experiment. Then, the pH value increased rapidly for 4 h until it reached pH 6.2. For the next 17 h, the pH slowly increased and plateaued at 6.4 (Figure 2c). Since Pacific salmon (Oncorhynchus nerka) is known to spoil faster than Atlantic salmon [27], we observed decreases in pH only for the first 2 h of the experiment. Then, the pH increased rapidly for 10 h until it reached pH 6.5 and remained the same for 18 h (Figure 2d). Although the absolute pH values were different, pH changes in tuna seemed to be similar with those of Atlantic salmon. Based on pH measurements, in fish, we define “fresh” for the initial 2 h when the pH started decreasing and “spoiled” after 12 to 13 h when the pH value plateaued at pH 6.4–6.5 for salmon and pH 6.0 for tuna, respectively. We termed “likely spoiled” between the time periods of “fresh” and “spoiled” when the pH values changed dramatically.

### 3.2. Staphylococcus Aureus and TBARS

The growth of Staphylococcus Aureus (S. Aureus), which is responsible for food poisoning through enterotoxin production, is frequently used as a biological indicator of food freshness [28]. 

**Figure 3:**
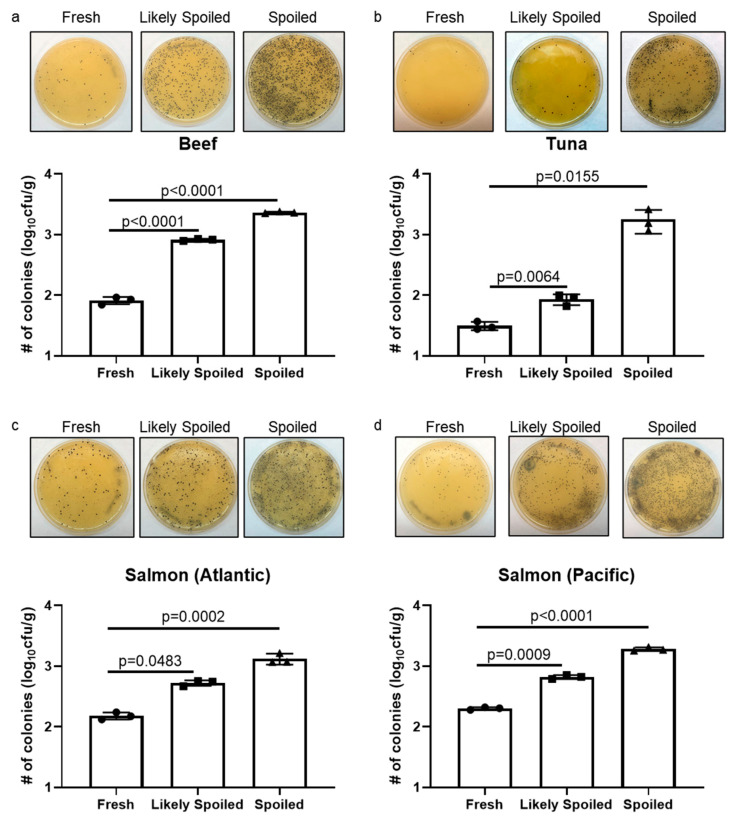
Growth of Streptococcus Aureus as an indicator of food freshness.

To support our pH data, we cultured S. Aureus from beef and fish samples at each time point representing states of food freshness. S. Aureus is Gram-positive bacteria causing food poisoning. Since it survives well in the harsh environment through the formation of biofilm, the growth of S. Aureus is found in a wide range of food [29]. After a 24-h incubation at 37 °C, the number of S. Aureus colonies on Baird Parker (BP) selective media plates (Millipore Sigma) was counted (Figure 3). In all four samples, we found that S. Aureus colonies were significantly increased at the time when food was “spoiled”. We also showed that significant changes in colony numbers when samples were “likely spoiled”, indicating microbiological changes in food. 

**Figure 4:**
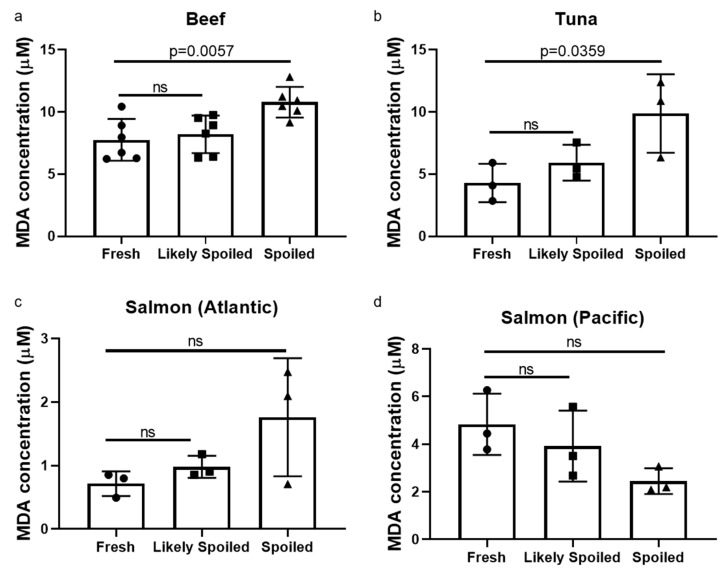
Thiobarbituric acid reacted substance (TBARS) assay to evaluate food freshness (ns: not significant).

To further determine food freshness using a biochemical method, lipid oxidation due to the degradation of fat from food samples was measured using thiobarbituric acid reactive substances (TBARS) assay [30]. Except for Pacific salmon, levels of malondialdehyde (MDA), a by-product of TBA activity, were significantly higher in the spoiled food samples (Figure 4). Although they did not meet statistical significance, there was a tendency of increases in MDA levels at the time point when food was “likely spoiled”.

### 3.3. Data Classification

**Figure 5:**
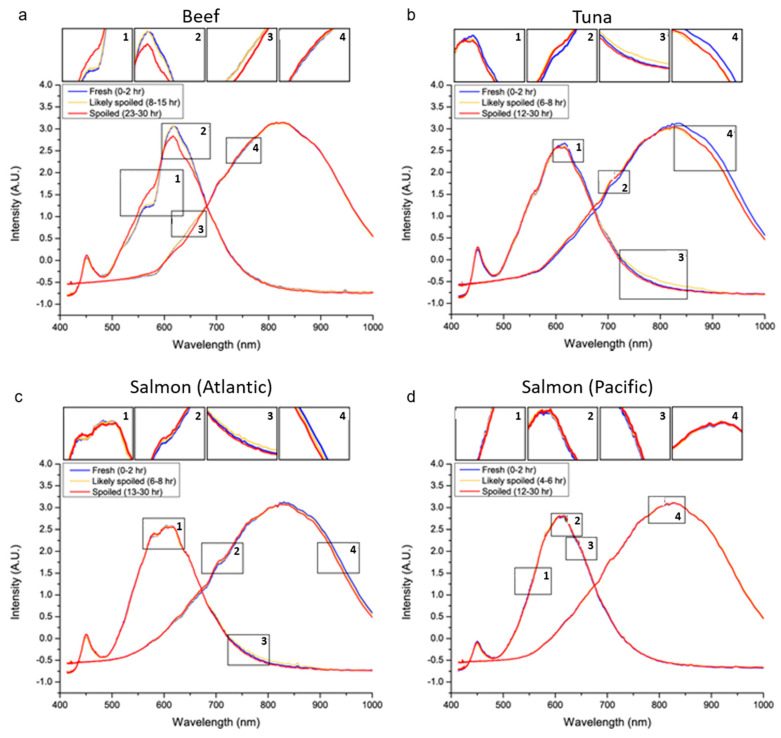
Spectral responses reflecting states of food freshness: (**a**) beef, (**b**) tuna, (**c**) Atlantic salmon, and (**d**) Pacific salmon. Average spectrum over 1000 spectra per each state after Standard Normal Variate (SNV) transformation are shown in the plots. Blue lines are for “Fresh”, yellow lines are for “Likely Spoiled”, and red lines for “Spoiled” samples. The lines with the maximum peak around 600 nm are the reflected spectrum of the LED light, and the lines with the peak around 800 nm are the reflected spectrum of the bulb light. For each plot, four areas with significant differences between the graphs are enlarged above for illustration purposes.

Spectral response data were automatically taken every minute using LinkSquare^®^ with two light sources: an LED and a bulb. These two light sources produced two spectrum data peaking around 600 nm (LED) and 900 nm (bulb). Since data were collected continuously, over 1000 graphs were averaged to show one spectral response data graph for each status. To minimize statistical errors, we kept the same number (approximately 1000 spectra per each category, as shown in Table 2) for each classification model by focusing on the dataset obtained from time points as color coded in Figure 5. 

Meat pigment consists of heme proteins such as hemoglobin and cytochrome C. Deoxyhemoglobin specifically promotes the oxidization of fat and darkens the color [31]. As shown in Figure 5a(1–3), spectral responses between 550 and 650 nm were different between “fresh” and “spoiled” beef, indicating color changes resulting from the oxidization of heme proteins. 

In both tuna and Atlantic salmon, we observed slight decreases in the intensity of bulb spectral response between 950 and 1000 nm (Figure 5(b4,c4)) when comparing “spoiled” samples to “fresh” ones. Since it is known that spectral responses in these wavelengths are highly influenced by the overtone band of water [32], the increased water content on the surface of “spoiled” samples seemed to drop the intensity of the spectral response. 

In salmon, carotenoids, astaxanthin, and canthaxanthin are pigments that absorb light under a 600 nm wavelength. Since these spectrum ranges are overlapped with absorbing regions of heme protein, which determine the freshness of salmon^33^, it is more useful to evaluate differences between 605 and 735 nm. Although changes were not explicit, there was a trend of increased intensities in both Atlantic and Pacific salmons when they were “spoiled” (Figure 5c,d).

Whether differences were prominent or not, these spectrum changes among “fresh”, “likely spoiled”, and “spoiled” were taken into consideration when applying to our machine learning algorithm for classification.

### 3.4. Validation

To analyze and classify the collected spectrum data, we divided them into two groups: the first group for training and the second group for verification. Data in both groups were collected independently to ensure the validity of the verification process. The first group was used for training to build a CNN-based classification model. To evaluate the accuracy of the model, we ran the obtained classification model with the second set for verification. The obtained confusion matrix is shown in Table 3. Rows in the table indicate the number of the spectral response from the known categories, and columns show three possible classification results that our model led to. Thus, the diagonal values of the confusion matrix represent the true positives of each class, while the others determine errors. The total accuracy can be calculated through the sum of diagonal values over all values. The total accuracy was 85% for salmon (84% for Atlantic salmon and 85% for Pacific salmon), 88% for tuna, and 92% for beef, indicating that the handheld VIS/NIR spectrometer with our CNN-based classification model can assess the freshness of food with high accuracy [33].

**Figure 6:**
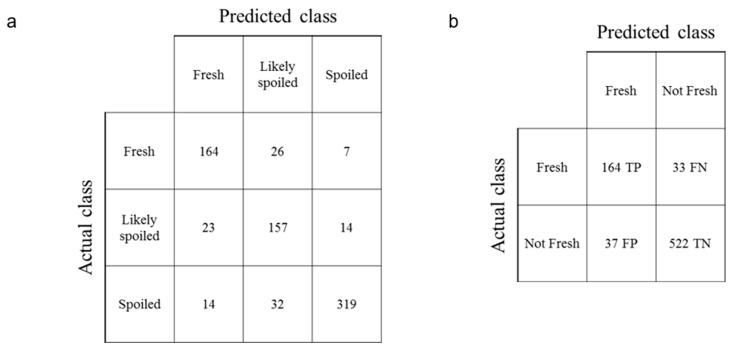
Confusion matrix (**a**) for a conventional two-by-two (2 × 2) matrix (**b**) of Salmon (Atlantic) “fresh”: TP is the True Positive of “fresh”, FP is the False Positive of “fresh”, FN is the False Negative of “fresh”, and TN is the True Negative of “fresh”.

The validation of our CNN-based machine learning algorithm can be more accurately explained by its sensitivity and specificity. Since our CNN-based classification model has three possible predictions, “fresh”, “likely spoiled”, and “spoiled”, it is necessary to change the current three-class classification test into three binary classification tests. For example, Figure 6a shows the confusion matrix of Atlantic salmon, which is the same as that in Table 1 The following Figure 4b is a modified two-by-two (2 × 2) confusion matrix assuming the binary classification asking if a spectral response of a sample is “fresh”. True Positive (TP) indicates the cases in which the actual “fresh” spectral responses are predicted correctly as “fresh”. False Positive (FP) indicates the cases in which the actual “not fresh” spectral responses are predicted incorrectly as “fresh”. False Negative (FN) indicates the cases in which the actual “fresh” spectral responses are predicted incorrectly as “not fresh”. True Negative (TN) indicates the cases in which the actual “not fresh” spectral responses are predicted correctly as “not fresh.” The sensitivity of the Atlantic salmon is 0.83 for “fresh” binary classification test, based on Equation (1). The specificity can be calculated similarly. When the sensitivity and specificity values are calculated for all three binary classification tests, Table 4 is obtained. It shows relatively high numbers, which also validates the possibility of using our CNN-based classification model.

## 4. Conclusions

We have developed a methodological approach to utilize a portable VIS/NIR spectrometer to assess the freshness of four food categories. The accuracy was 85% for salmon, 88% for tuna, and 92% for beef. Using the spectral response from a VIS-NIR portable spectrometer and a CNN-based machine learning-assisted classification, we have demonstrated a non-destructive, real-time measurement of food freshness. In addition, a CNN-based machine learning algorithm with a shift-invariant feature can minimize the effect of the variation caused using multiple devices in a real environment. We believe that this method can be easily applied to other foods and/or drinks, which will revolutionize the qualitative freshness measurement for consumers.

## Figures and Tables

**Table 1:** Multiple LinkSquare devices were used in this study, and we have labeled them from A to L (12 units). Each food samples were labeled as follows: Salmon Atlantic (SA), Salmon Pacific (SP), Tuna (T), and Beef (B). The number of 15, 15, 17, and 16 samples were used respectively for both training and verification spectra data.

	Sample	Salmon (Atlantic)		Salmon (Pacific)		Tuna		Beef	
Device									
		Training	Verify	Training	Verify	Training	Verify	Training	Verify
D1		SA1	SA4	SP1	SP4	T1		B1	
D2		SA2	SA5	SP2	SP5	T2, T3		B2, B3	
D3		SA3		SP3		T4		B4	B9
D4			SA6, SA7		SP6, SP7	T5	T9	B5	B10
D5			SA8, SA9		SP8, SP9	T6	T10, T11	B6	
D6			SA10, SA11		SP10, SP11	T7		B7	
D7			SA12		SP12	T8		B8	B11
D8			SA13		SP13		T12		B12, B13
D9			SA14		SP14		T13, T14		B14
D10			SA15		SP15		T15		B15
D11							T16		B16
D12							T17		
Total #		3	12	3	12	8	9	8	8

**Table 2:** The number of spectra collected for each food category.

	Salmon (Atlantic)	Salmon (Pacific)	Tuna	Beef
Fresh	1031	1055	840	1545
Likely spoiled	1042	1095	978	1803
Spoiled	1129	1457	1045	1694

**Table 3:** Confusion matrices of the machine learning models using the independently collected verification set.

(**a**) Salmon (Atlantic)				
	**Fresh**	**Likely Spoiled**	**Spoiled**	**Accuracy**
Fresh	164	26	7	83%
Likely spoiled	23	157	14	81%
Spoiled	14	32	319	87%
(**b**) Salmon (Pacific)				
	**Fresh**	**Likely Spoiled**	**Spoiled**	**Accuracy**
Fresh	73	9	4	85%
Likely spoiled	10	68	12	76%
Spoiled	3	7	136	93%
(**c**) Tuna				
	**Fresh**	**Likely Spoiled**	**Spoiled**	**Accuracy**
Fresh	153	4	9	92%
Likely spoiled	23	168	12	83%
Spoiled	8	29	293	89%
(**d**) Beef				
	**Fresh**	**Likely Spoiled**	**Spoiled**	**Accuracy**
Fresh	134	18	3	86%
Likely spoiled	20	259	2	92%
Spoiled	3	9	258	96%

**Table 4:** Calculation of sensitivity and specificity for four machine learning models. (**a**) Sensitivities show how well the models give correct answers for each class; (**b**) Specificities show how well the models reject the spectral response data from other classes. Both sensitivity and specificity are high enough to demonstrate the performance of machine learning models.

(**a**) Sensitivity				
	**Salmon (Atlantic)**	**Salmon (Pacific)**	**Tuna**	**Beef**
Fresh	0.83	0.85	0.92	0.86
Likely spoiled	0.81	0.76	0.83	0.92
Spoiled	0.87	0.93	0.89	0.96
(**b**) Specificity				
	**Salmon (Atlantic)**	**Salmon (Pacific)**	**Tuna**	**Beef**
Fresh	0.93	0.94	0.94	0.96
Likely spoiled	0.90	0.93	0.93	0.94
Spoiled	0.95	0.91	0.94	0.99
